# The effects of music intervention on burn patients during treatment procedures: a systematic review and meta-analysis of randomized controlled trials

**DOI:** 10.1186/s12906-017-1669-4

**Published:** 2017-03-17

**Authors:** Jinyi Li, Liang Zhou, Yungui Wang

**Affiliations:** 1 Department of Humanities and Social Sciences, The Third Military Medical University, Chongqing, China; 2 Research Institute of Field Surgery, Daping Hospital, The Third Military Medical University, Chongqing, China; 3 The Third Military Medical University, Chongqing, 400038 China

**Keywords:** Music intervention, Burn patients, Pain, Anxiety, Meta-analysis, Systematic review

## Abstract

**Background:**

The treatment of burn patients is very challenging because burn injuries are one of the most severe traumas that can be experienced. The effect of music therapy on burn patients has been widely reported, but the results have been inconsistent. Thus, we performed a systematic review and meta-analysis of randomized controlled trials in burn patients to determine the effect of music during treatments.

**Methods:**

We searched a variety of electronic databases, including MEDLINE (via PubMed), EMBASE, Cochrane Library, Psychinfo, VIP Database for Chinese Technical Periodicals (VIP) and China National Knowledge Infrastructure (CNKI) for relevant trials on the basis of predetermined eligibility criteria. from their first available date through February 2016. Our search focused on two key concepts: music interventions (including music, music therapy and music medicine) and physical activity outcomes (including pain, anxiety, burn characteristics, dressing changes, wound care, debridement and rehabilitation). Two reviewers independently screened records and extracted data from all eligible studies. Statistical heterogeneity was determined using Q-test and the *I* ^2^ statistic. The endpoints included standardized mean differences (SMDs) and 95% confidence intervals (CIs). Publication bias was tested by Begg’s funnel plot and Egger’s test.

**Results:**

A total of 17 studies met the inclusion criteria, for a total of 804 patients. A statistically significant difference in pain relief was demonstrated between music and non-music interventions (SMD = −1.26, 95% CI [−1.83, −0.68]), indicating that music intervention has a positive effect on pain alleviation for burn patients. The results indicated that music interventions markedly reduced anxiety in individuals compared to non-music interventions (SMD = −1.22, 95% CI [−1.75, −0.69]). Correspondingly, heart rate decreases were found after treatments that included music interventions (SMD = −0.60, 95% CI [−0.84, −0.36]).

**Conclusion:**

In summary, a positive correlation was found between treatments including music interventions and pain alleviation, anxiety relief, and heart rate reduction in burn patients. However, additional high-quality studies with carefully considered music interventions for burn patients are still needed.

**Electronic supplementary material:**

The online version of this article (doi:10.1186/s12906-017-1669-4) contains supplementary material, which is available to authorized users.

## Background

The treatment of burn patients is very challenging because burn injuries are one of the most severe traumas that can be experienced. As medical technology has advanced, the majority of burn patients are now being successfully resuscitated and typically undergo early escharotomy, skin transplantation, and antibiotic administration in addition to receiving nutritional support, which together dramatically decrease their mortality rate [1]. However, burn patients must still experience many painful procedures, including skin grafting, escharectomy, debridement, dressing changes and physical rehabilitation. Burn patients usually face a series of physiological and psychological problems during treatment. Pain is a major problem and occurs during all stages of treatment. The adequate management of pain may make recovery more tolerable and affect morbidity by means of prevention of elevated metabolism, thereby reducing the chance of malnutrition and deterioration of the immune system [2]. And the researchers question the safety of analgaesics and anxiolytics in patients with major burns because of their requirement for massive fluid resuscitation has the potential for contributing to hemodynamic instability [3]. Moreover, the use of sedation and analgesia must be limited in pediatric burn patients. There is a very close relationship between anxiety and pain [4], and anxiety is the most common emotional issue faced by burn patients, as reported in early studies [5, 6]. As such, the treatment of burn patients must incorporate a holistic view of pain and anxiety. Effectively adjusting treatment parameters to manage pain and anxiety is necessary for burn patients throughout treatment.

The use of music interventions in the clinic has a long history. These interventions have typically been used during treatment and rehabilitation. To date, many studies have reported the use of music as an intervention during dental procedures, surgery, chemotherapy, and injections [7–12]. Music interventions have also been used to manage pain and anxiety in patients during medical procedures for many years. A study reported by Bradt showed the effects of music interventions on preoperative anxiety in surgical patients [8]. Furthermore, studies conducted by Chlan et al. have shown that the use of music interventions can reduce anxiety in ICU patients on mechanical ventilation [13, 14]. Other authors have also demonstrated anxiety reduction in mechanically ventilated ICU patients through music interventions [15–17]. Wang et al. [18] has indicated that the use of music interventions can significantly improve pain score, anxiety, heart rate, arterial pressure, and satisfaction score for patients undergoing a variety of endoscopic procedures. Notably, Hole et al. [19] demonstrated that music can help alleviate postoperative pain, anxiety, and analgesia needs in addition to improving patient satisfaction during recovery.

Music as an intervention has wide applicability during burn treatment. Particularly as a form of complementary and alternative medicine (CAM), music therapy has been widely used in multiple clinical fields due to its non-pharmacological, non-invasive and easily accessible features. The study of music interventions for burn patients began in the late 1970s. Christenberry published the first paper regarding the application of music therapy for burn patients and outlined a corresponding protocol for the intervention [20].

Music intervention is widely used during dressing changes and debridement to help decrease pain and anxiety in burn patients. The majority of past studies have indicated that music has positive effects with regard to the alleviation of pain for burn patients, especially non-severe pain [4, 21–27]. In addition, Robb et al. [28] found that music assisted relaxation and decreased anxiety in burn patients and increased their compliance during debridement and dressing changes. However, some discrepancies exist regarding the outcomes produced by the clinical application of music therapy for pain management. Ferguson [4] studied the effects of relaxing music on perceived levels of pain and anxiety during range-of-motion exercises and found that the music produced no significant effects on pain relief. Furthermore, there were no significant differences between pretest and post-test anxiety scores following the music intervention. Another study published in 2006 showed that the effects of music interventions on pain and anxiety in pediatric patients during donor-site dressing changes were not conclusive [29]. Thus, the effects of music intervention remain unclear and require further investigation. In previous studies, the primary types of music intervention investigated have included music therapy and music medicine. Music therapy is an interpersonal process during which professional staff who have completed an approved music therapy program use music and all of its facets—physical, emotional, mental, social, aesthetic, and spiritual—to help clients accomplish individualized goals [30]. Music medicine involves relatively passive listening to pre-recorded music offered by a researcher or clinician without the involvement of a music therapist or a defined therapeutic process.

Few reviews have been reported regarding the use of music interventions for burn patients; indeed, only two studies [31, 32] have reviewed the effects of music therapy on burn patients, and these two studies were not meta-analyses. Other studies have reviewed the effects of non-pharmaceutical therapy, which is not restricted to music therapy or music medicine on burn patients [3, 33]. No review study thus far has conducted a meta-analysis examining the effects of music interventions on burn patients. The purpose of the current systematic review and meta-analyses was to evaluate the effects of randomized controlled trials (RCTs) of music interventions for burn patients during treatment procedures and to provide recommendations for future research and clinical practice.

## Methods

### Search strategy

The study was designed in accordance with the Cochrane Handbook for Systematic Reviews of Interventions. Our results were reported according to the Preferred Reporting Items for Systematic Reviews and Meta-Analysis statement [34]. We performed a search of all literature regarding the clinical application of music therapy on burn patients using the following databases: MEDLINE (via PubMed), EMBASE, Cochrane Library, Psychinfo, VIP and CNKI. We searched the databases from their earliest available dates through February 2016. Both MESH terms and free text words describing ‘the use of music interventions (including music therapy and music medicine)’ and ‘the measurement of physical activity outcomes (including pain, anxiety, burn characteristics, dressing changes, wound care, debridement and rehabilitation) were used in the search. The articles of these two sets were then combined using the Boolean ‘AND’ operator. The search builders were presented as follows: ‘music’ or ‘music intervention’ or ‘music therapy’ or ‘music medicine’ AND ‘burn’ or ‘burn patient*’ or ‘burn pain’ or ‘burn anxiety’ or ‘dressing changes’ or ‘debridement’ or ‘wound care’ or ‘burn rehabilitation (Additional file 1: Table S1). Reference checking and citation tracking of the included articles were manually performed to identify additional studies meeting the inclusion and exclusion criteria. In addition, we manually searched the Chinese databases of journals, dissertations and magazines for related articles as well as the references to these articles.

### Inclusion and exclusion criteria

The inclusion criteria were RCTs with a parallel group, crossover or cluster design that included burn patients undergoing various procedures (e.g., dressing changes, debridement, range of motion exercises, and surgery). The subjects in the intervention group received music intervention before and/or during and/or after procedures, whereas the subjects in the control group underwent procedures without music. The music interventions included music therapy and music medicine. The music could be live music or recorded music, and the styles of music were not limited. Studies were excluded if their raw data could not be extracted or if music was not the main intervention method used during treatment, such as interventions that involved music combined with massage. Each article should be scored according to the Cochrane Collaboration’s tool for assessing risk of bias and those less than 2 points should be excluded. Literature studies written in English and Chinese have been included in this manuscript.

### Data extraction

Primary outcome measurement in this meta-analysis was pain intensity, while anxiety was considered a secondary outcome measurement. Data were carefully and independently extracted from all eligible studies by two investigators (JL, ZL according to the inclusion criteria mentioned above using a prespecified Microsoft Excel spreadsheet. The extracted data included study characteristics (e.g., author name, year of publication, sample size, patient age, total body surface area (TBSA) and type of music), effect measurements (e.g., pain score, level of anxiety, and heart rate), and quality indicators (e.g., adequate sequence generation, allocation concealment, and blinding). Disagreement was resolved by discussion or consulting with a third reviewer (YW).

### Risk of bias assessment

The methodological quality of the studies was independently evaluated by two investigators (JL, ZL) according to the Cochrane Risk of Bias tool for RCTs [35]. Any differences were resolved by consulting with a third reviewer (YW).

### Statistical analysis

Statistical heterogeneity was determined using Q-test and the *I* ^2^ statistic. For cases in which *P* ≤ 0.10 and *I* ^2^ ≥ 50%, a random effects model was applied. Otherwise, a fixed effects model was used. The endpoints were SMDs and 95% CIs. Publication bias was assessed using Begg’s funnel plot and Egger’s test.

## Results

### Study selection

**Fig. 1:**
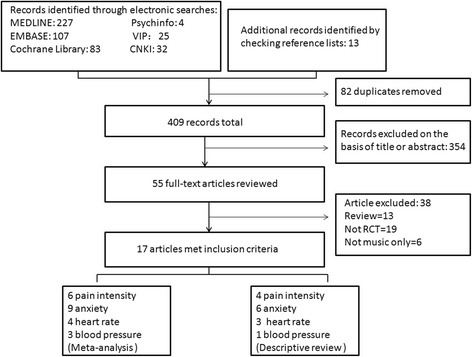
PRISMA flow diagram: study selection

After performing an extensive electronic search combined with a manual search, 491 records were identified, resulting in an initial library of 409 references following the removal of 82 duplicates. 354 records were excluded on the basis of title or abstract. Fifty-five full-text articles reviewed to determine its eligibility for inclusion and exclusion criteria. After an independent review of titles and abstracts, 38 records were excluded for failing to meet the inclusion criteria. A total of 17 RCTs were included in the final review (Fig. 1). The following variables were extracted from the included studies: length of study, size of trial sample, ages and genders of participants, and procedures and intervention methods used. These data are shown in Table 1.

**Table 1:** Characteristics of the included studies

	Sample *(treatment / control)*	Age	Gender *(male/female)*	TBSA_(A)_ % *(range)*	Procedure	Interventions						Duration of music	Measurement tools_(D)_
						Technique_(B)_	Music	Selection	Intervention description_(C)_	Control	Other		
Miller et al. (1992) [43]	17 (9/8)	40.9/27.8 *(mean treatment/control)*	16 M, 1 F	1–39%	Dressing change	Muralvision	Recorded music	Investigator-selected music	①③	Placebo effect	Medication	During procedure	MGPQ, STAI
Robb et al. (1995) [28]	20 (10/10)	8–20	N/A	N/A	During preoperative period	MAR	Recorded music	Self-selected music	①②③④	Usual care	Medication	Before and during procedure	STAIC
Fratianne et al. (2001) [26]	25	7–83	16 M, 9 F	1–43%	Debridement	MBI & MAE	Live music	Patient’s preferred music	①②③④⑤	Usual care	Medication	Before, during and after procedure	WBFRS, VAS, TOMRI
Haythronthwaite et al. (2001) [44]	42	43.6 *(mean)*	32 M, 10 F	3–65%	Dressing change	Music distraction	Recorded music	Self-selected music	①③⑤	Sensory focusing, usual care	Medication	20 min before and during procedure	11-LS, BDI, Burn-CSQ
Ferguson et al. (2004) [4]	11 (5/6)	18–75	8 M, 3 F	7–50%	Range of motion	Music relaxation	Recorded music	Self-selected music	①⑤	Usual care	Exercise	During procedure	VAS, STAIC, H-PCMS
Chen Shujuan et al. (2005) [39]	40 (20/20)	23–54	40 M	12–49%	Debridement process	Music medicine	Recorded music	Investigator-selected music	①③	Usual care	No	Twice a day for 30 min each time; 30 days for a course of treatment	HAMA, HRSD
Whitehead-Pleaux et al. (2006) [29]	14 (8/6)	6–16	5 M, 9 F	N/A	Dressing change	Music therapy	Live music	Self-selected music	①②③④	Verbal interaction	No	During procedure	NAPI, WBFRS, FT
Lin Huiting et al. (2007) [36]	40 (20/20)	20–55	40 M	13–50%	Debridement process	Music medicine	Recorded music	Patient’s preferred music	①⑤	Usual care	No	During procedure	VAS
Tan et al. (2010) [24]	29	8–71	24 M, 5 F	3–40%	Debridement process	MBI & MAE	Live music and recorded music	Patients’ music preferences	①②③④⑤	Usual care	Medication	Before, during and after procedure	VAS, MTIS
Liu Chenyuan et al. (2010) [22]	120 (60/60)	8–86	69 M, 51 F	N/A	Dressing change	Music medicine	Recorded music	Patient’s preferred music	①④	Usual care	No	20 min before and during procedure	VAS, STAI
Liang Wanling et al. (2010) [42]	62 (31/31)	17–50	45 M, 17 F	N/A	Isolation area	Music medicine	Recorded music	Self-selected music by patient/ family	①③	Usual care	No	Patient-selected music played for 1 h at 7:00 and 17:00	SAS, SDS
Yang Yong (2011) [38]	46 (23/23)	36 *(mean)*	26 M, 20 F	N/A	During hospitalization	Music medicine	Recorded music	Self-selected music from list	①③④	Usual care	No	Twice a day for 20–30 min each time	VAS, SDS
Zhang Qian et al. (2012) [23]	60 (30/30)	19–50	29 M, 31 F	4–5%	Cold therapy	Music medicine	Recorded music	Investigator-selected music	①⑤	Usual care	Cryotherapy	During procedure	VAS, STAI
Jiang Mingzhu (2013) [41]	64 (32/32)	19–63	43 M, 21 F	Ocular	During hospitalization	Music medicine	Recorded music	Investigator-selected music	①③	Usual care	No	At 9:00 and 15:00 each day for 30–60 min each time	SAS
Ren Yue et al. (2014) [37]	72 (36/36)	N/A	N/A	20–60%	Dressing change	Music medicine	Recorded music	Nurse-selected music	①⑤	Usual care	Medication	15 min before and during procedure	SAS
Zhou Tao (2014) [40]	42 (21/21)	47.2/45.1 *(mean treatment/control)*	23 M, 19 F	N/A	Daily nursing care	Music medicine	Recorded music	Investigator-selected music	①③④	Usual care	No	Before and during procedure	SAS, SDS
Najafi et al. (2015) [25]	100 (50/50)	31.08/31.18 *(mean treatment/control)*	62 M, 38 F	6–48%	During hospitalization	Music intervention	Recorded music	Patient’s preferred music	①③⑤	Usual care	Medication	Music intervention was offered once a day (20 min) for 3 consecutive days	VAS

*Abbreviations*: *MGPQ* McGill Pain Questionnaire (including PPI and PRI; *PPI* Present pain intensity, *PRI* Pain rating index), *WBFRS* Wong/Baker Faces Rating Scale, *NAPI* The Nursing Assessment of Pain Index, *STAI* The Spielberger’s State-Trait Anxiety Inventory, *BDI* The Beck Depression Inventory, *VAS* Visual analog scale, *HAMA* Hamilton Anxiety Scale, *HRSD* Hamilton Rating Scale for Depression, *11-LS* 11-point Likert scales, *STAIC* The State-Trait Anxiety Index for Children, *FT* The Fear Thermometer, *TOMRI* Trippett Objective Muscle Relaxation Inventory, *MTIS* The Muscle Tension Inventory Scale, *H-PCMS* Hewlett-Packard Component Monitoring System, *SAS* Self-Rating Anxiety Scale, *SDS* Self-Rating Depression Scale

(A) TBSA: Total body surface area. (B) Techniques. Muralvision: A distraction-relaxation music therapy technique combining video or pictures with music for distraction. MAR (music-assisted relaxation): This method includes music listening, deep diaphragmatic breathing, progressive muscle relaxation, and imagery. MBI (music-based imagery): The MBI component occurred in the patient’s room for 15 to 30 min before and after the procedure and provided relaxing and safe experiences to the patient through music listening. MAE (musical alternate engagement): The MAE intervention was used to provide more physically engaging activities and participatory musical tasks during dressing changes in the treatment area. (C) Intervention description①Music intervention form (music medicine or operational process);②Technique introduction (if the techniques of music intervention has been introduced or not); ③Procedure description (Start time, End time, operational process);  ④Materials and Settings (Instruments, stereo equipment, environment); ⑤Music characteristics (style, genre, tempo, volume, et al.). (D) Measurement tools

### Study characteristics

The current review included 804 burn patients from 17 RCTs comparing patients undergoing treatments with and without music interventions. Table 1 lists characteristics from all included studies. These trials included nine studies published in Chinese [22, 27, 36–42] and eight studies published in English [4, 24, 25, 28, 29, 43, 44]. The ages of the included patients ranged between 6 and 86 years old. Five studies reported the average age of their patients. From a total of 17 literatures, two literatures with 92 patients didn’t provide gender information. There is a total of 722 patients in the rest 15 literatures, among them, 67.6% were male patients. The types of procedures investigated included dressing change [22, 29, 37, 43, 44], debridement [24, 26, 36, 39], preoperative procedures [28], range of motion rehabilitation [4], cold therapy [23], daily nursing care [40], isolation [42] and hospitalization [25, 37, 38].

Most of the music used in the intervention was self-selected by the patient [4, 28, 29, 38, 42, 44] or based on a patient’s preferences [22, 24–26, 36]. Recorded music was used in 15 studies, and live music was used in three studies [24, 26, 29] (Table 1). The main methods used for music intervention in the included trials were attention distraction methods such as Muralvision or musical alternate engagement (MAE) and relaxation methods such as music-assisted relaxation (MAR) and music-based imagery (MBI) (Table 1).

### Outcome measurements

Pain intensity was assessed in ten studies [4, 22–26, 29, 36, 43, 44] using the following measurement tools: the Visual Analogue Scale (VAS) [4, 22–25, 36], the Wong/Baker Faces Rating Scale (WBFRS) [26, 29], the McGill Pain Questionnaire (MGPQ) [43], the Nursing Assessment of Pain Index (NAPI) [29] and the 11-point Likert scales (11-LS) [44]. In one study, pain intensity experienced by pediatric patients was assessed using the WBFRS and the NAPI [29] (Table 1). Only six studies were included in this meta-analysis; the remaining four studies were descriptively reviewed because data could not be extracted from them.

Fifteen studies assessed anxiety descriptors [4, 22–26, 28, 29, 37–43] using the following measurement tools: State-Trait Anxiety Inventory forms (STAI) [4, 22, 23, 28, 43], the Self-Rating Anxiety Scale (SAS) [37, 40–42], the Hamilton Anxiety Scale (HAMA) [39], the Fear Thermometer (FT) [29] and the VAS [24–26, 38] (Table 1). However, six studies were excluded due to a lack of raw data; therefore, only nine studies were included in our analysis of anxiety descriptors (Table 1).

Blood pressure was evaluated based on measurements of systolic blood pressure (SBP) and diastolic blood pressure (DBP). Four studies reported the effects of music intervention on SBP and DBP [22, 25, 28, 41]; of these, three were included in the meta-analysis [22, 25, 41].

Heart rate, another continuous variable in terms of vital signs, was extracted in four studies and combined in the meta-analysis [22, 25, 29, 41]; of these, three studies provided only descriptive reviews and were not included in the meta-analysis [26, 28, 37].

### Risk of bias

**Fig. 2:**
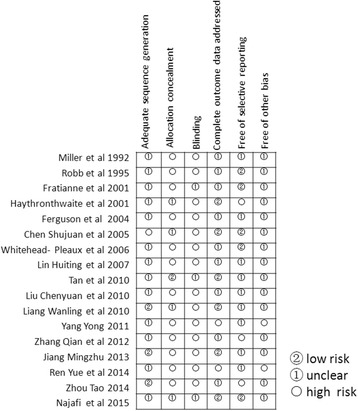
Results of bias risk assessments

To assess the risk of bias, the patients were randomly allocated into two groups; however, the majority of included studies did not describe their exact methods of randomization [4, 22–26, 28, 29, 36–39, 43, 44]. Only three studies claimed that allocation was based on the generation of a random number table [40, 41] or random lottery [42]. The blinding of treatment allocation was obtained by concealed envelopes in one study [24]. Because of the nature of music intervention, the evaluation criteria used for double-blinding were obscure for most studies. Blinded methodology was used as much as possible in the included studies. In one study, the physicians treating the patients were blinded regarding whether the patients were listening to music prior to treatment [26]. In Najafi et al. study, a blinded co-researcher recorded and measured the experimental data [25]. In another study, to decrease rater bias, the research nurses were not assigned to the patients in the research group prior to the study [24]. In addition, two of the studies did not provide any measurement raw data but just final results and therefore demonstrated possible outcome-reporting bias [37, 38] (Fig. 2).

### Outcomes of meta-analysis

**Fig. 3:**
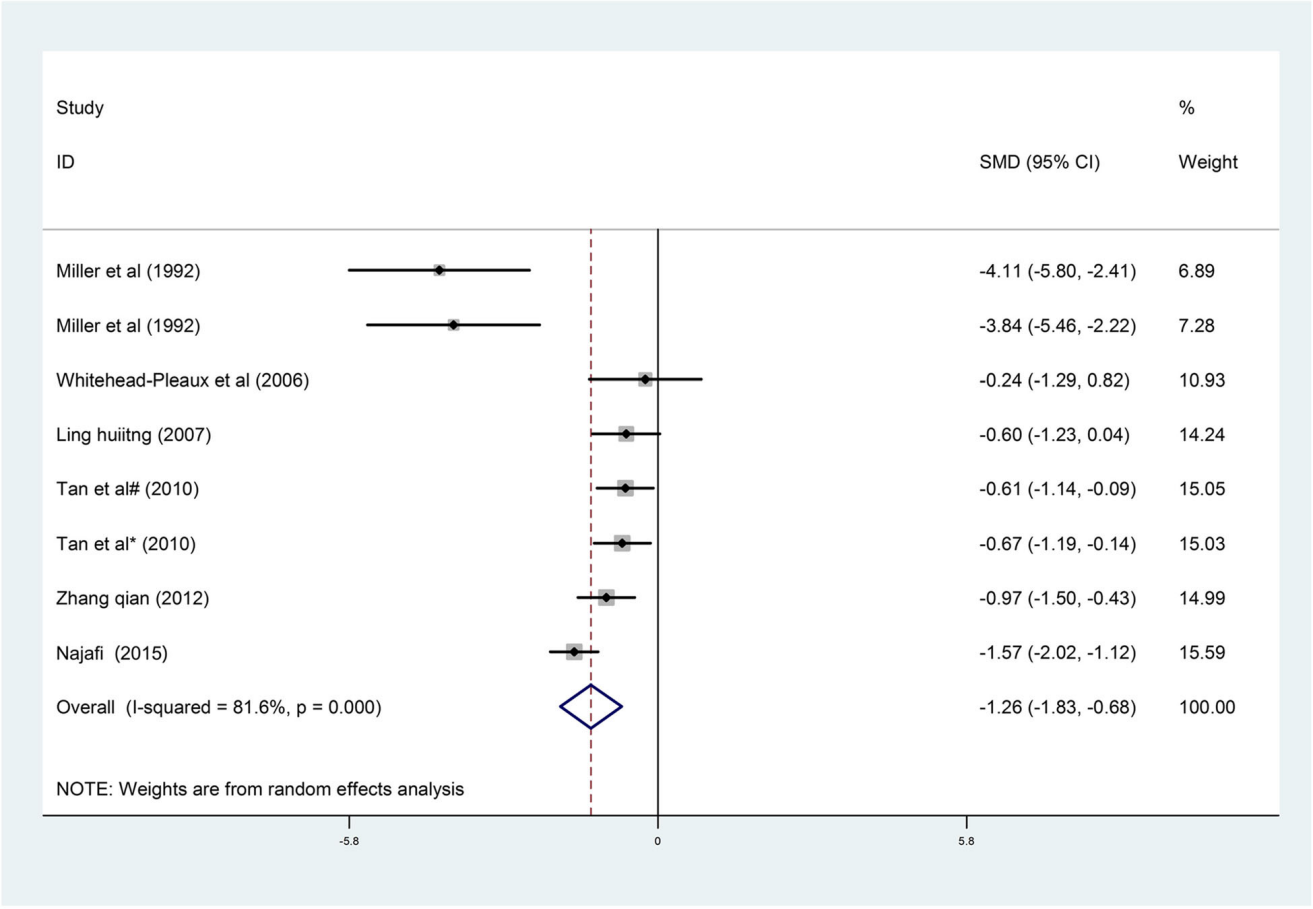
Forest *plot* of music therapy for burn patients during treatment procedures, outcome parameter: pain
  Four studies were included in descriptive reviews. In Fratinanne et al. [26] study, self-reported pain was improved in the music therapy group by over four intervals during treatment procedures. The self-reporting of pain was significantly decreased for those who received music therapy compared to those who did not. Liu Chenyuan et al. [22] study reported that 98.33% of patients had level 0 or level 1 pain during dressing changes in the experimental group, while only 80% of patients in the control group had similar low pain levels. The majority of patients in the control group had significantly higher pain levels than those in the experimental group during dressing changes. However, contrary evidence was reported in other studies. Haythronthwaite et al. found that patients in a sensory focusing group experienced greater pain relief than those in a music distraction group based on serial pain ratings [44]. In Ferguson’s study, although there was a difference between pretest and post-test pain across groups, no difference in pain was found between the groups [4].
- Secondary Outcomes
*Anxiety Level*. The included anxiety scores demonstrated statistically significant heterogeneity (*I* ^2^ = 87.0%, *P* < 0.001). The results showed a statistically significant reduction in the anxiety levels of the burn patients (SMD = −1.22, 95% CI [−1.75, −0.69]) in the intervention group compared to those in the control group [23–26, 29, 36–39, 41–43] (Fig. 4).

- Primary Outcome
*Pain*. The meta-analysis of six trials and 260 burn patients for measures of pain intensity demonstrated significant heterogeneity *(I* ^2^ = 81.6%, *P* < 0.001). The pooled result from the random effects model demonstrated significant differences in pain scores between the music intervention group and the non-music intervention group (SMD = −1.26, 95% CI [−1.83, −0.68]) [23–25, 29, 36, 43] (Fig. 3). Music intervention was found to reduce the pain experienced by burn patients during treatment procedures.

**Fig. 4:**
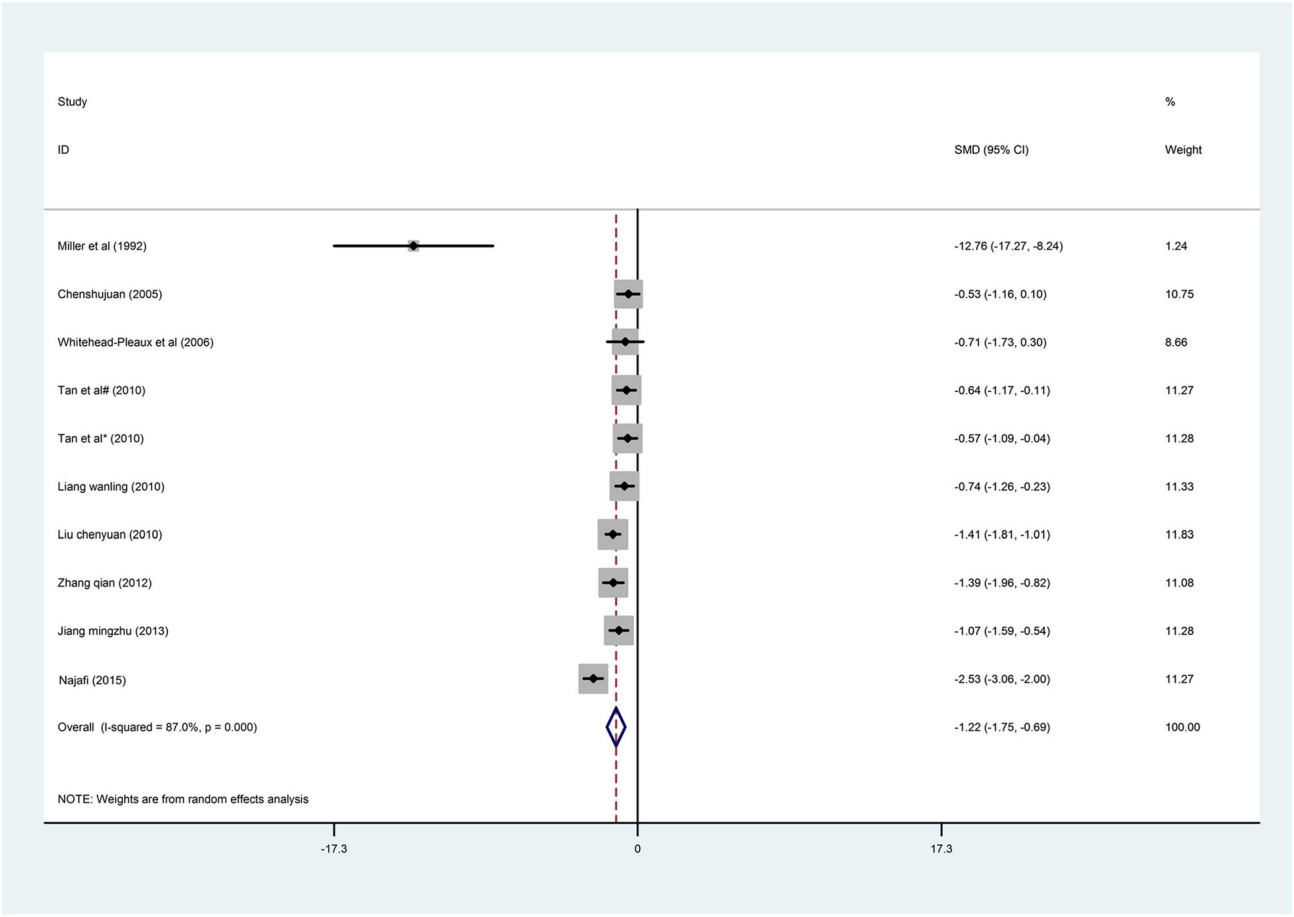
Forest *plot* of music therapy for burn patients during treatment procedures, outcome parameter: anxiety
  Although the study reported by Robb et al. [28] did not include sufficient data to be included in the meta-analysis, a significant decrease in anxiety scores was found for the experimental group compared to the control group. Zhoutao reported that music intervention had a significant positive effect on anxiety alleviation; the effective ratio of the control group was 9.52%, whereas the effective ratio of the experimental group was 52.38% (*P* < 0.05) [40]. Although two studies that were conducted in China were not included in the meta-analysis due to a lack of pretest raw data, the results of these studies also indicated that music interventions significantly reduced anxiety for severe burn patients [38] during hospitalization or during dressing change when combined with anesthetics [37]. Fratinanne et al. [26] indicated that self-reported anxiety during medical procedures was reduced by four intervals in the music therapy group, but no statistical significance was observed. Moreover, Ferguson and Voll also reported that no significant reduction in anxiety was found during therapy including music relaxation [4].
*Heart Rate*. The effects of music intervention on heart rate during burn treatment procedures were extracted from four studies in the meta-analysis [22, 25, 29, 41], and the statistical heterogeneity for this variable was significant (*I* ^2^ = 88.8%, *P* < 0.001). Compared with the usual care group, heart rate was significantly decreased in the music intervention group (SMD = −0.60, 95% CI [−0.84, −0.36]) (Fig. 5).

**Fig. 5:**
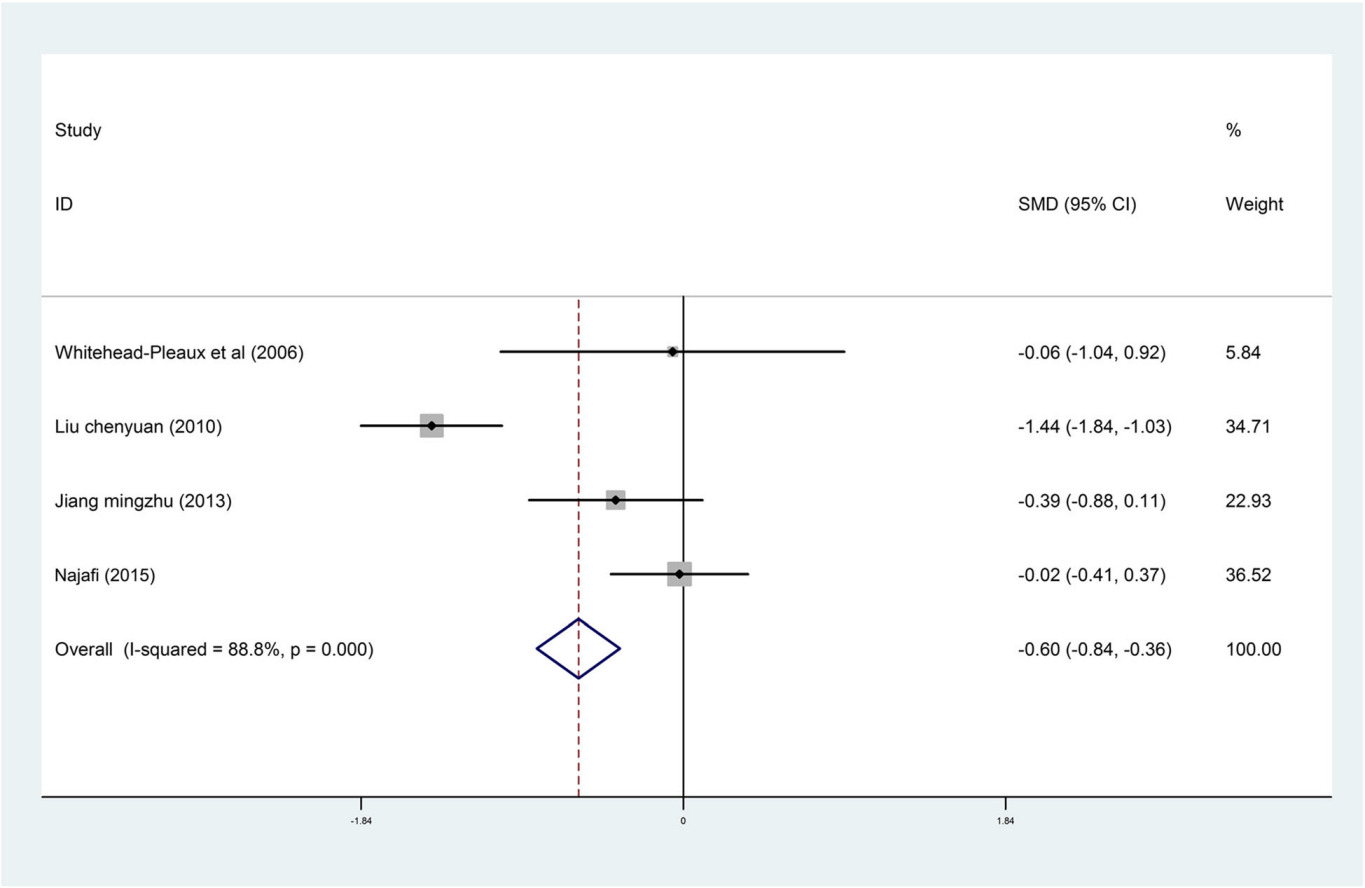
Forest *plot* of music therapy for burn patients during treatment procedures, outcome parameter: heart rate
  Three studies that had reported the effects of music interventions on heart rate were not included in the meta-analysis due to ineligibility. Robb et al. [28] indicated that music interventions showed no significant effect on heart rate between pre- and post-test periods for either group. Frantianne et al. [26] reported that music therapy had a slight effect on heart rate, although the difference was not significant. However, in Renyue et al. [37] study, the post-test results revealed that music interventions decreased heart rate significantly during dressing changes compared to the control group.
*Blood Pressure*. Four studies reported on the effects of music interventions on blood pressure [22, 25, 28, 41]; of these, three were included in the meta-analysis. The random effects pooled result did not demonstrate differences between the intervention group and the control group with regard to blood pressure during treatment procedures (SBP: SMD = −0.37, 95% CI [−1.18, 0.45]; DBP: SMD = −0.24, 95% CI [−0.68, 0.20]) [22, 25, 41] (Figs. 6 and 7). Similarly, Robb et al. [28] study found no significant differences in heart rate between the pre- and post-test period for either group.

**Fig. 6:**
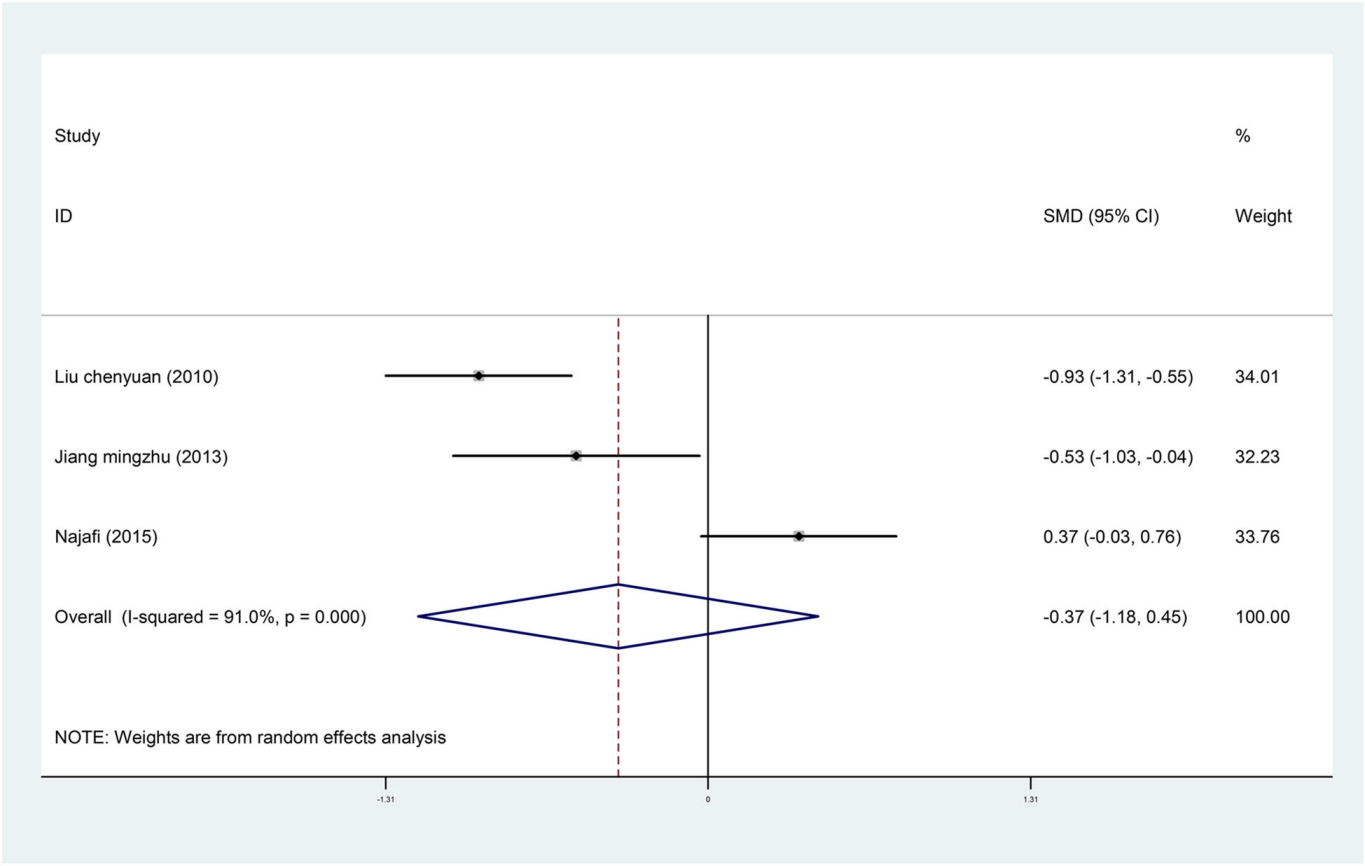
Forest *plot* of music therapy for burn patients during treatment procedures, outcome parameter: SBP

**Fig. 7:**
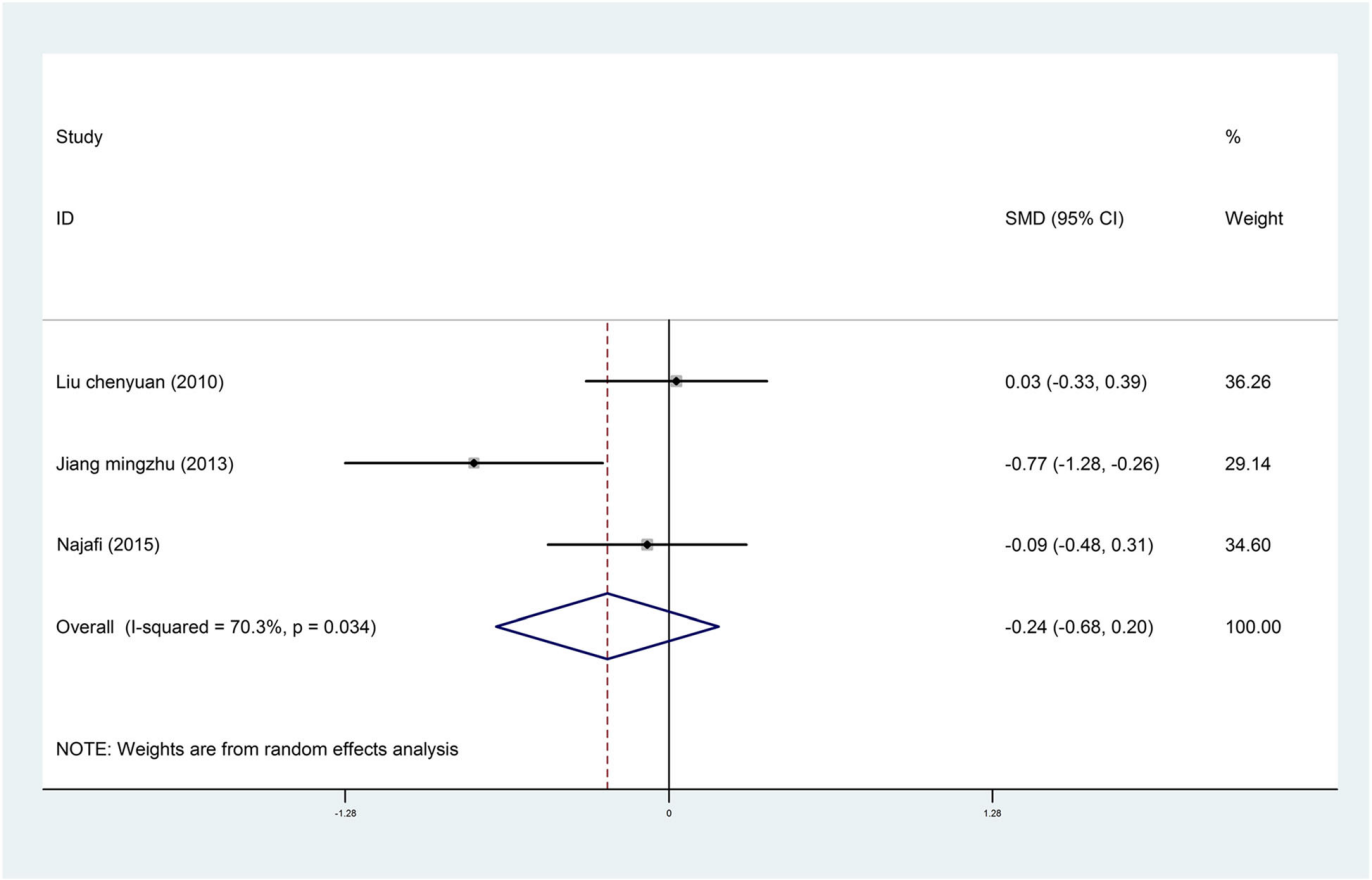
Forest *plot* of music therapy for burn patients during treatment procedures, outcome parameter: DBP
*Respiration Rate*. Two of the four studies that included information regarding the effect of music therapy on respiration rate showed statistically significant differences between pre- and post-treatment measurements of respiratory rate across the groups [4, 25]. The other two studies showed no significant difference in respiration between groups during the preoperative period or during dressing changes [28, 29].

### Publication bias

Publication bias was estimated using Begg’s test (for pain, z = −1.43, *P* = 0.202; for anxiety, z = 0.36, *P* = 0.721) and Egger’s linear regression test (for pain, z = 1.11, *P* = 0.266; for anxiety, *t* = −1.18, *P* = 0.271). The results suggested that there was no significant evidence of publication bias (Additional file 2: Figure S1). Furthermore, the results of sensitivity analysis indicated that the overall results in the meta-analysis were robust and reliable.

## Discussion

The purpose of the current systematic review was to evaluate the effects of music interventions on burn patients undergoing medical procedures.

During burn treatment and nursing, many factors cause patients to experience pain, including the wound itself, dressing changes, bathing, debridement, excision and grafting. Furthermore, escharotomy, plastic surgery, routine occupational therapy, physical therapy, nursing care and rehabilitation therapy also induce pain. In the current systematic review and meta-analysis, music was used as an intervention in seven different types of procedures, including dressing change, debridement, range of motion exercise, preoperative preparations, cold therapy, nursing care and isolation. The effects of music intervention in other clinical fields, including for critically ill patients receiving mechanical ventilatory support [13] and coma patients [45], has been well studied. These studies provide a reference for the application of music therapy for burn patients in the ICU.

The current meta-analysis showed a significant decline in pain intensity before and after patients received music interventions. The majority of studies showed that music had a positive effect on pain relief; the burn patients exposed to music typically reported low to moderate amounts of pain during treatment. This result is consistent with results from studies of the use of music for the relief of chronic, non-severe pain [46]. However, one of the studies included in the current systematic review and meta-analysis showed that music therapy not only reduced chronic pain but also severe pain [24]. A prospective, randomized crossover clinical trial was conducted in an inpatient burn unit using MBI and MAE. The study indicated that music therapy decreased pain, anxiety, and muscle tension in burn patients during acute procedures [24]. So far, Tan’s findings were different than that of Fratianne [26] and Prensner [47]. Therefore, larger sample studies regarding the effect and application field of MBI & MAE are needed in the future.

It is well known that the most widely accepted neurological principle underlying the mechanism for the association of music and pain relief is the gate control theory of pain reported by Melzack and Wall [24, 29, 48]. This theory asserts that stimulation by non-noxious input is able to suppress pain. However, a recent study reported that music therapy modulates pain perception through at least two different mechanisms that involve changes in the activity of delta and gamma bands at different stages of pain processing [49]. These results provided novel insights into the neurological principals that underlie the achievement of pain relief following music therapy. It is necessary to find a solution to resolve the pain and anxiety felt by burn patients undergoing treatment procedures.

The use of music as an intervention has shown the potential to reduce pain during burn treatment. Haythronthwaite’s study indicated that the effect of music on pain relief was more obvious in a sensory focusing group compared with a music distraction group [44]. It is worth noting that the music intervention methods used in the music distraction group fell under the purview of medicine rather than music therapy. Thus, if rigorous music therapy methods were introduced into this research according to treatment target, they may produce the same effects as those observed in the sensory focusing group. Tan’s study also proved this point [24]. More studies are needed to reveal the roles of music intervention, especially music therapy, in severe pain control. However, based on the above-referenced study by Hauck et al. [49], the combination of relaxation and distraction with music intervention may help patients cope with pain. Although individual studies have shown that submitting burn patients to music interventions provides some evidence of decreased pain intensity and anxiety, there have been no indications that any specific type of music offers more benefits.

Consistent with previous studies, our study believed that it is vital to establish appropriate standard protocols of music therapy during different burn treatment procedures [32]. In this study, four kinds of music therapy protocols including Muralvision, MAR, MBI and MAE have been used in these included studies. One literature which was not included in this Meta-analysis introduced the effect of other music therapy protocols including Song Phrase Cued Response (SPCR)、Adapted Progressive Muscle Relaxation (APMR)、MBI and the Relaxation Response Elicitation (RRE) during burn treatment. Those different protocols have been adapted to meet the specific needs of burn patients during specific procedures [47]. However, further researches are still needed to provide evidence-based clinical practice of music therapy protocol for patients with specific needs.

Furthermore, in most studies, the music was selected by patients from existing music lists or was the patient’s own preferred music. However, in Chinese studies, music interventions have mainly relied on music medicine. In China, the quality of music intervention still needs improvement due to the lack of professional music therapists and standardized training. The improvement of professional music therapy may promote future research into music therapy in China.

Eleven studies reported significant anxiety relief between the intervention group and the control group. Correspondingly, patient satisfaction also improved during treatment in three studies [37, 38, 42]. However, the results of these trials differed from those in other studies in the review, and they did not provide enough raw data on specific indicators to support the meta-analysis or answer our email requests for more detailed data [4, 44].

More appropriate measurement scales and methods for relieving pain and anxiety are needed for burn patients. In the 17 studies included here, many different scales were used to measure pain and anxiety. The VAS, the MGPQ, the WBFRS and the NAPI were used to measure pain, while the STAI, the VAS, the FT, the HAMA and the SAS were used to measure anxiety. However, Tan’s research showed that the graphic rating scale, the MGPQ and the STAIR are not suitable for the study of burn patients since these measurements are all subjective self-reports. However, this point of view is still controversial Furthermore, Whitehead-Pleaux et al. [29] found that the FT could not capture the effects of music on pain and anxiety on pediatric patients undergoing painful procedures due to their limited understanding of the terms.

Meanwhile, we found that the application of analgesic during burn treatment has received more and more attentions [24, 26, 47]. One study demonstrated a positive correlation between burn patient increased comfort levels when music therapy was used in conjunction with pharmaceutical treatments [32]. However, no data could be extracted from the included literatures regarding the effect of music therapy on analgesic use. Thus, further studies are needed to investigate the effect of music therapy on pain medication during burn treatment. In addition, burn patients not only faced physical pain, but also psychological distress. Therefore, to establish a physical-psychological intervention program becomes necessary for burn patients. The current systematic review and meta-analysis is the first to assess the effectiveness of music interventions on burn patients undergoing treatment. However, the study results should be interpreted in light of its limitations, most of which are related to the original trials. First, in the majority of the 17 included studies, the risk of bias was moderate. The overall trial quality was reduced due to the lack of concealed allocation or blinded therapists assessing outcome measures. Second, the sample sizes in most of the trials were small. Third, there was heterogeneity in the types of patient populations studied, types of music interventions applied, and types of treatment used. Although heterogeneity existed among the studies, the standardized mean differences per group were calculated, and the results for the pain and anxiety intensity outcomes were pooled. In addition, we would have attempted to adjust for the heterogeneity by performing a subgroup analysis or a meta-regression analysis, but the number of studies was insufficient to perform these analyses. Moreover, some of the studies lacked quantitative measurements of specific indicators, making their inclusion in the meta-analysis risky. However, we included these study results in the review to avoid potential bias.

## Conclusions

In conclusion, our study presents limited evidence from 17 individual trials that burn patients may experience cumulative benefits from music interventions in terms of decreased pain and anxiety, leading to better treatment prognosis. Music intervention has a positive effect on pain alleviation, anxiety reduction and heart rate control, which provides evidence to support the advantages of its use during burn treatment. Further high-quality studies with carefully considered music interventions are recommended to provide more solid evidence on both the short-term and long-term effects of this intervention strategy on burn patients.

## Additional files

Additional file 1: Table S1. Search strategy. (DOC 31 kb)
Additional file 2: Figure S1. Begg’s funnel plot and Egger’s linear regression test. (A) Begg’s funnel plot for pain. (B) Begg’s funnel plot for anxiety. (C) Egger’s linear regression test for pain. (D) Egger’s linear regression test for anxiety. (JPG 237 kb)

## Abbreviations

11-LS: 11-point Likert scales
CAM: Complementary and alternative medicine
CIs: Confidence intervals
CNKI: Chinese National Knowledge Infrastructure
CqVip: Chongqing Vip of Chinese Science and Technology Periodical Database
DBP: Diastolic blood pressure
FT: Fear Thermometer
HAMA: Hamilton Anxiety Scale
*I*^*2*^: I-square
MAE: Musical alternate engagement
MAR: Music-assisted relaxation
MBI: Music-based imagery
MGPQ: McGill Pain Questionnaire
NAPI: Nursing Assessment of Pain Index
RCTs: Randomized controlled trials
SAS: Self-Rating Anxiety Scale
SBP: Systolic blood pressure
SMDs: Standardized mean differences
STAI: State-Trait Anxiety Inventory forms
TBSA: Total body surface area
VAS: Visual Analogue Scale
WBFRS: Wong/Baker Faces Rating Scale
