# Supplementary material for: The effects of music intervention on burn patients during treatment procedures: a systematic review and meta-analysis of randomized controlled trials
Source: BMC Complement Altern Med. 2017 Mar 17;17:158. doi: 10.1186/s12906-017-1669-4 (PMC5356403; doi:10.1186/s12906-017-1669-4)
Supplement: Additional file 1: Table S1. — Search strategy. (DOC 31 kb) [file 12906_2017_1669_MOESM1_ESM.doc]

**Table S**1. The Search Strategy of Meta-analysis

| Database | Search Strategy |
| --- | --- |
| MEDLINE (via PubMed) | ((((music) OR music intervention) OR music therapy) OR music medicine) AND ((((((burn) OR burn patient) OR dressing change) OR debridement))) NOT( burns [Author])  ((((music) OR music intervention) OR music therapy) OR music medicine) AND (burn) AND ((((pain) OR anxiety) OR wound care) OR rehabilitation)) NOT( burns [Author])  N=227 |
| EMBASE | ((((music) OR music intervention) OR music therapy) OR music medicine) AND (((((((((burn) OR burn patient) OR burn pain) OR burn anxiety) OR dressing change) OR debridement) OR wound care) OR burn rehabilitation))  N=107 |
| Cochrane Library | ((((music) OR music intervention) OR music therapy) OR music medicine) AND (((((((((burn) OR burn patient*) OR burn pain) OR burn anxiety) OR dressing change) OR debridement) OR wound care) OR burn rehabilitation [title/abstract/key/ words])))  N=83 |
| Psychinfo | ((((music) OR music intervention) OR music therapy) OR music medicine) AND ab ((burn) OR burn patient) OR burn pain) OR burn anxiety) OR dressing change) OR debridement) OR wound care) OR burn rehabilitation))  N=4 |
| VIP | ((((music) OR music intervention) OR music therapy) OR music medicine) AND ((((((burn) OR burn patient) OR dressing change) OR debridement)))  ((((music) OR music intervention) OR music therapy) OR music medicine) AND (burn) AND ((((pain) OR anxiety) OR wound care) OR rehabilitation))  N=25 |
| CNKI | ((((music) OR music intervention) OR music therapy) OR music medicine) AND (((((((((burn) OR burn patient) OR burn pain) OR burn anxiety) OR dressing change) OR debridement) OR wound care) OR burn rehabilitation))  N=32 |

Note: VIP: VIP Database for Chinese Technical Periodicals, CNKI: China National Knowledge Infrastructure.

Search was performed in all fields of the databases including MEDLINE (via PubMed), EMBASE, VIP Database for Chinese Technical Periodicals（VIP）and China National Knowledge Infrastructure (CNKI).

Search was performed in title, abstract and key words of Cochrane Library.

Search was performed in abstract of Psychinfo database.

The variety of electronic databases were searched from their first available date through February 2016.
