# Supplementary figures and images for: The effects of music intervention on burn patients during treatment procedures: a systematic review and meta-analysis of randomized controlled trials
Source: BMC Complement Altern Med. 2017 Mar 17;17:158. doi: 10.1186/s12906-017-1669-4 (PMC5356403; doi:10.1186/s12906-017-1669-4)

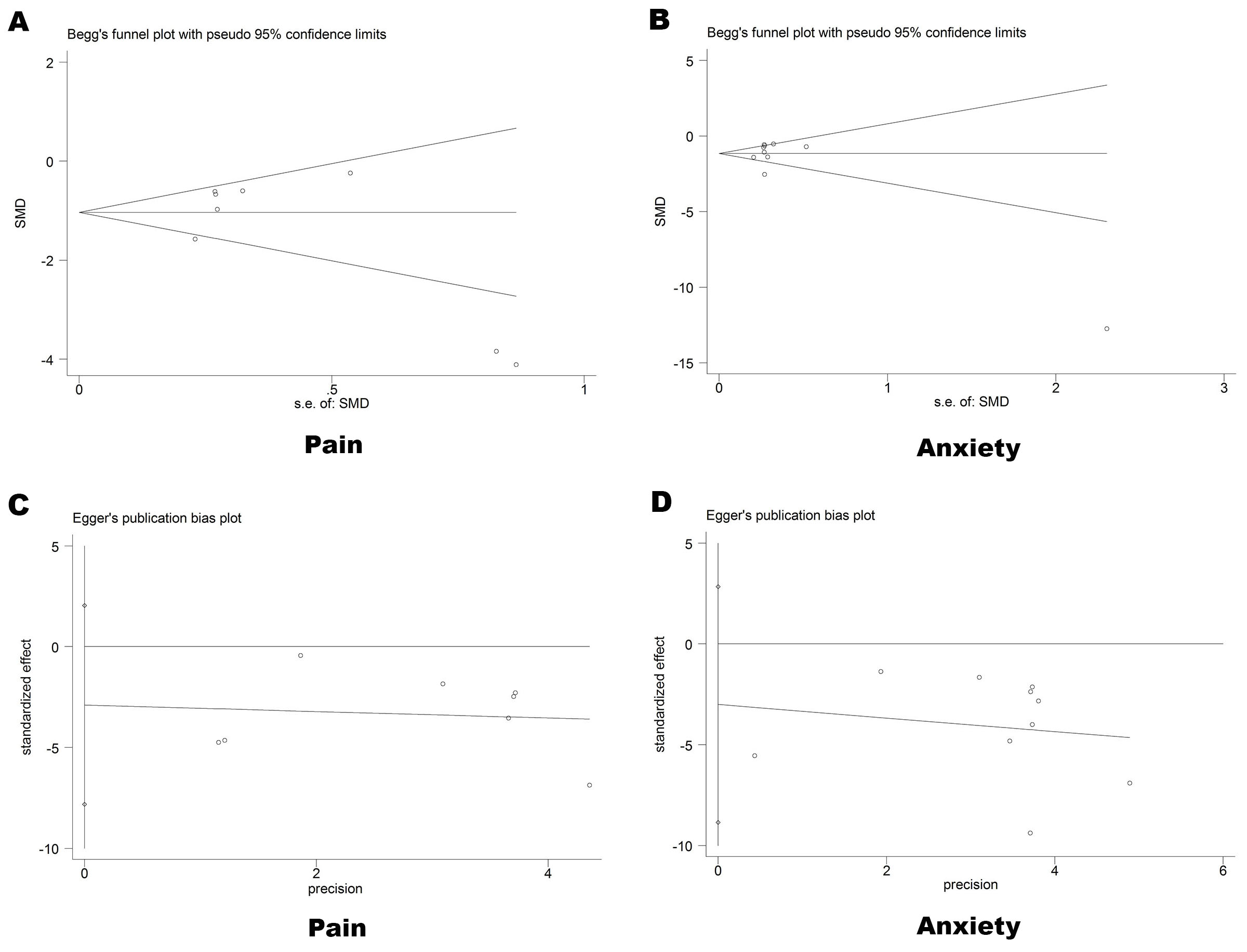

Supplement: Additional file 2: Figure S1. — Begg’s funnel plot and Egger’s linear regression test. (A) Begg’s funnel plot for pain. (B) Begg’s funnel plot for anxiety. (C) Egger’s linear regression test for pain. (D) Egger’s linear regression test for anxiety. (JPG 237 kb) [file 12906_2017_1669_MOESM2_ESM.jpg]
